# “Keeping Moving”: factors associated with sedentary behaviour among older people recruited to an exercise promotion trial in general practice

**DOI:** 10.1186/s12875-015-0284-z

**Published:** 2015-05-28

**Authors:** Ruth Heseltine, Dawn A. Skelton, Denise Kendrick, Richard W. Morris, Mark Griffin, Deborah Haworth, Tahir Masud, Steve Iliffe

**Affiliations:** Department of Primary Care & Population Health, University College London, Royal Free Campus, Rowland Hill St., London, NW3 2PF UK; School of Social and Community Medicine, University of Bristol, Canynge Hall, 39 Whatley Road, Bristol, BS8 2PS UK; Division of Primary Care, University of Nottingham, Floor 13, Tower Building, University Park, Nottingham, NG7 2RD UK; School of Health & Life Sciences, Glasgow Caledonian University, Cowcaddens Rd, Glasgow, Lanarkshire G4 0BA UK; Nottingham University Hospital NHS Trust, Derby Rd, Nottingham, NG7 2UH UK

**Keywords:** Older people, Physical activity, Sedentary behaviour, Exercise promotion

## Abstract

**Background:**

Sedentary behaviour is detrimental to health, even in those who achieve recommended levels of physical activity. Efforts to increase physical activity in older people so that they reach beneficial levels have been disappointing. Reducing sedentary behaviour may improve health and be less demanding of older people, but it is not clear how to achieve this. We explored the characteristics of sedentary older people enrolled into an exercise promotion trial to gain insights about those who were sedentary but wanted to increase activity.

**Method:**

Participants in the ProAct65+ trial (2009–2013) were categorised as sedentary or not using a self-report questionnaire. Demographic data, health status, self-rated function and physical test performance were examined for each group. 1104 participants aged 65 & over were included in the secondary analysis of trial data from older people recruited via general practice. Results were analysed using logistic regression with stepwise backward elimination.

**Results:**

Three hundred eighty seven (35 %) of the study sample were characterised as sedentary. The likelihood of being categorised as sedentary increased with an abnormal BMI (<18.5 or >25 kg/m^2^) (Odds Ratio 1.740, CI 1.248–2.425), ever smoking (OR 1.420, CI 1.042–1.934) and with every additional medication prescribed (OR 1.069, CI 1.016–1.124). Participants reporting better self-rated physical health (SF-12) were less likely to be sedentary; (OR 0.961, 0.936–0.987). Participants’ sedentary behaviour was not associated with gender, age, income, education, falls, functional fitness, quality of life or number of co-morbidities.

**Conclusion:**

Some sedentary older adults will respond positively to an invitation to join an exercise study. Those who did so in this study had poor self-rated health, abnormal BMI, a history of smoking, and multiple medication use, and are therefore likely to benefit from an exercise intervention.

**Trial registration:**

ISRCTN reference: ISRCTN43453770

## Background

Sedentary behaviour (SB), conventionally defined as low energy-expenditure activity undertaken in a sitting or reclining position [1], is associated with adverse physical and mental health outcomes [2]. Sedentary behaviour appears to have deleterious health effects even where physical activity recommendations are met [3], and so sitting time is now recognised as a health risk factor independent of physical activity [4–6]. Older adults are most likely to be sedentary [7, 8].

Long periods of sitting are associated with a bigger waist circumference, depression and social isolation, and an increased risk of death [2]. Sedentary older adults are more likely to have the metabolic syndrome [9, 10], type 2 diabetes [3, 9, 11], cardiovascular disease [3, 9, 12], depression [13], lower bone mineral density [14], greater co-morbidity [13] and higher all-cause mortality [3, 15] than less sedentary older adults. Increased sedentary behaviour is further associated with functional limitations [11, 13, 16], falls [13], poorer quality of life [17], experiencing severe pain [16] and lower likelihood of successful aging, measured across both physical and psychological domains [17]. Since the health risks are significant and far reaching, understanding the characteristics of sedentary individuals is potentially important in targeting health interventions to reduce sedentary behaviour.

Epidemiological studies have described the characteristics of sedentary older people. Increasing sedentary behaviour is associated with older age [11, 16, 18], abnormal BMI [9, 12, 16, 18–20], higher waist circumference [11], smoking [11, 12], living alone [13, 19], being unmarried [11, 12], lack of full-time employment [19] and lower levels of social support [16]. Occasionally the associations are conflicting. Sedentary behaviour has been shown to be more prevalent in women [9, 16], men [11, 12], neither sex [18], in those with lower education [9, 11, 12, 16, 18, 19], higher education [13], lower income [18] and higher income [13].

A small qualitative study by Chastin et al. [21] sheds light on the determinants, motivators and barriers older women express in relation to reducing sitting time. They attributed their sedentary behaviour to pain (predominantly musculo-skeletal), variable daily energy levels, external pressure from family and friends to undertake sitting activities and societal stereotypes of older people [21]. They also felt an entitlement to sit in older age, failed to recognise its objective harms and felt a sense of wellbeing from social sedentary activities [21]. Motivators to activity included pain relief (after sedentary periods), the necessity of household chores, in order to be useful to those around them and to relieve boredom & depression [21]. They also identified environmental barriers to increasing activity including lack of standing activities for older people, poor weather, and lack of public resting places outside the home [21]. However, they felt that more community-based opportunities to be active would help them reduce their sedentary behaviour [21]. Whilst sometimes perceived as a hard to reach group, promotion of appropriately tailored exercise in older adults may prove both acceptable and effective in reducing sedentary behaviour.

To reduce sedentary behaviour we must first quantify it. Although challenging, several studies have quantified SB in older people [19, 22–24]. Whilst younger adults are engaged in SB during 60 % [25] of their waking hours, older adults have been shown objectively (using accelerometry) to be sedentary more than 70 % of the time [22, 24], for around 8–10 h of the waking day, and this increases linearly with age [22, 26]. Conversely, self-reported SB is typically underestimated by as much as 50 % [23, 27]. Espana-Romero et al. [28] showed that older people both overestimate their physical activity and underestimate their sedentary behaviour; men by 26 % and women by 34 % amounting to a difference of 4–6 h/day. Sedentary behaviour is thus commonplace in older adults and under-estimated by self-report.

This discrepancy between objective & self-reported measures is explained by Van Uffelen et al. who demonstrate that older adults made judgements and generalisations when answering physical activity questionnaires [29]. Older people had difficulty in generating examples of sedentary activities beyond those explicitly listed. They were uncertain whether non-leisure sedentary activities should be included as sedentary (eating, driving etc.). They also generalised to a ‘typical day’ rather than giving a contemporaneous report of the day’s activities [29]. Nonetheless, six activities have been shown to correlate best with SB; napping, reading, listening to music, watching TV, having a hobby and talking to friends [30]. These can be used to estimate total sedentary time. The underestimate in self-reporting appears to correlate in a linear fashion with objectively measured sedentary behaviour [30]. Therefore, it is reasonable to measure SB using self-reported questionnaires like PASE (Physical Activity Scale for the Elderly) and adjust for under-reporting.

Understanding the volume of sedentary behaviour in older people and the negative associations with health leads to questions over the validity of physical activity targets. Guidelines focus on the attainment of moderately-vigorous physical activity (MVPA) [4], even though older adults spend as little as 1 % of their waking day in MVPA [22]. Exercise promotion for older adults should perhaps also aim at reducing SB [31] and displacing inactivity into light physical activity such as household chores, slow walking or light gardening [6]. These changes can increase the metabolic rate and energy expenditure markedly [32] and are associated with better physical health in adults aged 65+ [31]. Breaks to sedentary time are independently & beneficially associated with lower waist circumference, BMI, triglyceride concentration and 2-h plasma glucose [33]. Reduction & displacement of SB could be a useful target for older people’s health promotion.

However, the literature on modifying sedentary behaviour is limited. Fitzsimons et al. [34] demonstrated an objective reduction in sedentary activity and an increase in activity in a small group of older adults following a motivational interview about reducing sedentary behaviour. Gardiner et al. found an objective reduction in sedentary time after a single-session of goal setting [35]. Magistro et al. [36] found that functional fitness improved in a group of sedentary older adults who undertook a 4 month small-group walking exercise programme. These smaller exploratory studies suggest that change is possible. However, when Stevens et al. [37] conducted a meta-analysis of activity-based interventions in general practice, only 6 suitable studies were found which were “heterogeneous and difficult to replicate or standardise”. Further work is required to establish the effectiveness of interventions to reduce sedentary behaviour in older adults.

This study is novel because it explores the extent of sedentary behaviour in participants in an exercise intervention trial aimed at older people (65 and over) and carried out in general practice, and describes the characteristics associated with sedentary behaviour. The research questions were:1) Do sedentary older people join an exercise study? 2) What demographic, functional and health factors are associated with sedentary behaviour in this self-selected population of older people?

## Methods

### Participants

Data from the ProAct65+ trial were used in this study [38, 39]. ProAct65+ was a pragmatic 3 arm parallel design cluster controlled trial of class-based exercise (Falls Management Exercise Programme, FaME), home based exercise (Otago Exercise Programme, OEP) and usual care (treatment as usual, TAU) amongst community-dwelling UK residents aged 65 years and over. The primary outcome was the proportion of participants meeting recommended levels of physical activity (30 mins of MVPA 5 days/week) 12 months after cessation of the intervention phase of the trial (FaME, OEP, TAU). The participants were interviewed and surveyed at regular intervals (baseline, 6, 12, 18, 24 months post-randomisation) over a 2 year period (2009–2011). Ethical approval was obtained and consent details can be viewed in the full trial document [39].

**Fig. 1:**
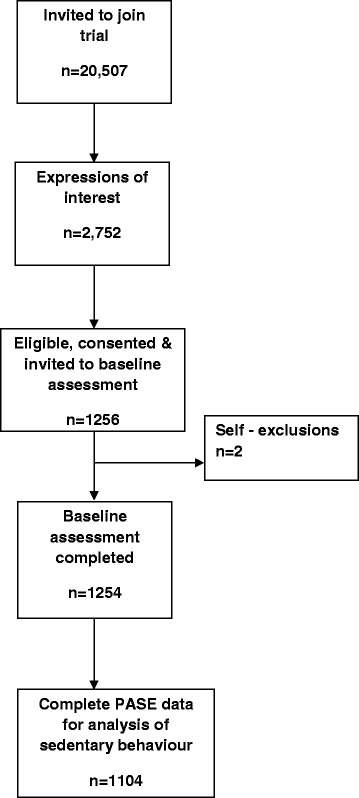
Derivation of the sample for analysis of sedentary behaviour

General Practices were recruited in Nottingham, Derby and London, through the Primary Care Research Network. GPs screened for, and a researcher verified, eligible participants who were 65 years and over, independently mobile indoors and outdoors (with or without walking aid) and physically able to participate in group exercise classes. Exclusion criteria were already meeting recommended MVPA activity targets, having 3 or more falls in the previous year, unstable clinical conditions, inability to safely follow exercise instructions, not living independently, already receiving long term physiotherapy or receiving palliative care. Participants received an invitation letter from their usual GP. Of 20,507 people approached, 2752 adults expressed interest and 1256 gave consent to join the study [38]. One participants dropped out before attending for the baseline assessment and one subsequently withdrew all data from the study, leaving a sample of 1254. Complete PASE data was available for 1104, and this sample was used for the analysis of sedentary behaviour. Figure 1 shows the derivation of the sample.

### Data collection

Multiple assessments were made at baseline. The measures considered in this study were selected according to the literature (see above), are listed below and described in detail elsewhere [38]. They consisted of: demographic data, functional assessments, health status and self-rated function, as shown in Table 1.

**Table 1:** Data sets obtained from ProAct65+ participants

Demographic Data	Objective Functional assessments	Self-rated Health status function
Age	30 s chair rise	Everyday activity limitation
Gender	Timed get-up & go test (TUG)	Comorbidities
Body Mass Index (BMI)	Functional reach	Medication usage
Smoking status	Modified Clinical Romberg (FICSIT)	Informal home help
Household composition		Current level of physical activity
Education data		PHQ (Physical Health Questionnaire)
Household income		Perceived physical & mental health (SF-12)
Employment status		Older People’s Quality of Life Questionnaire (OPQOL)

### Sedentary behaviour

The dependent variable was sedentary behaviour (SB). An estimate measure of sedentariness was determined using two questions in the PASE questionnaire: “over the past 7 days, how often did you participate in sitting activities such as reading, watching TV or doing handicrafts?” & “On average, how many hours per day did you engage in these sitting activities?” Since older people underestimate sedentary behaviour by self-report by up to 50 % our operational definition of sedentariness was those who reported sitting-based activities for over 4 h on more than 5 days per week, corresponding to the 8+ hours of objective sedentariness found by Espano-Romero and colleagues [28]. The non-sedentary group reported sedentary behaviour for fewer than 4 h/day on fewer than 5 days/week.

### Variable characterisation

Category choices were determined by the characteristics associated with sedentary behaviour in epidemiological studies. Each examined variable was dichotomised as shown in Table 2, where possible applying standards from previous studies. Where no literature standard existed we dichotomised pragmatically. For example, smoking status was split into never- or ever- smokers since the majority of smokers had already quit but may have significant prior lifetime exposure. BMI was divided into normal or abnormal (encompassing both over- (>25 kgm^2^) & under-weight (<18.5 kgm^2^) groups) as only a very small number of participants were underweight which we took to represent poor health. Age was dichotomised to capture the distinction between the ‘younger’ (65–74) and ‘older’ (75 and over) old.

**Table 2:** Description of characteristics examined

*Characteristic*	*Groups*	
*CODED*	*0*	*1*
Sedentary	No	Yes (sitting >4 h/day, > 5 days/week)
Demographic Data		
Age	65–74	75 years and over
Gender	Male	Female
BMI	Normal	Not normal (<18.5, >25)
Smoking status	Never	Ever (current/ex)
Household composition	Living as a couple (married/co-habiting)	Not living as a couple (alone/extended family/other)
Highest Educational Level	Higher (FE & Uni)	School only
Household pre-tax income/annum (GBP)	Categorical continuous	
Employment status	Employed	Unemployed
Functional Assessments		
30-s Chair stand [42]	Continuous	More stands = better function
TUG (falls risk) [43]	Continuous	Faster = better function
Functional reach (cm) [44]	Continuous	Further = better function
FICSIT balance scale	Continuous	Higher score = better balance
Health Status & Self-rated function		
Activity limitation (days/month)	Continuous	0–31 days/month
Number of comorbidities	Continuous	Integer values
Number of medications	Continuous	Integer values
Informal home help	Absence	Presence
Current level of activity	Some	None
Easy public transport use (PHQ)	Yes	No
Use of walking aid (PHQ)	No	Yes
Any falls in the past year (PHQ)	No	Yes
SF-12-PCS [45–47]	Continuous	Higher score = better health
SF-12-MCS [45–47]	Continuous	Higher score = better health
Lubben social network	Continuous	
OPQOL [48]	Continuous	Higher score = better quality of life
FESI [49]	Continuous	Higher score = more falls concern

Continuous variablets included 30-s chair stand, timed up & go test in seconds, functional reach, FICSIT (Frailty and Injuries: Cooperative Studies of Intervention Techniques) score (balance), activity limitation, number of comorbidities, number of medications, quality of life and SF12 scores (physical and mental component scores).

### Data analysis

Baseline characteristics of ProAct65+ participants were compared with their sedentary behaviour status using chi-squared univariable analyses for dichotomised variables and logistic regression for continuous variables. Backwards stepwise elimination logistic regression analysis (Wald) was chosen as suitable for an exploratory study in a large sample, and was used to adjust for correlations between characteristics that were significant on univariable analysis. Results are presented as unadjusted and adjusted Odds Ratios with 95 % confidence intervals.

## Results

Three hundred eighty seven of the 1104 participants (35 %) were sedentary at baseline. Table 3 shows the associations between participant characteristics and sedentary behaviour, with each association presented unadjusted and adjusted for all other characteristics.

**Table 3:** Unadjusted odds ratios showing associations between continuous and dichotomised variables and sedentary behaviour

Continuous variable	Description	Non-sedentary: mean (s.d.)	Sedentary: mean (s.d.)	OR	Lower CI	Upper CI	*P*-value
SF12_PCS^a^ *n* = 1049	Total score OR for each extra point	37.54 (5.98) *n* = 664	35.91 (7.21) *n* = 385	0.962	0.943	0.980	<0.001
No. of medications *n* = 1047	OR for each additional medication	3.74 (3.20) *n* = 663	4.49 (3.26) *n* = 384	1.073	1.032	1.115	<0.001
No. of comorbidities *n* = 1053	OR for each additional comorbidity	1.93 (1.54) *n* = 666	2.34 (1.61) *n* = 387	1.174	1.086	1.271	<0.001
Activity limitation *n* = 1054	Self-reported no. of days limited per month OR for each additional day limited	1.01 (4.60) *n* = 667	2.09 (7.10) *n* = 387	1.033	1.010	1.056	0.004
Timed Up & Go (TUG) *n* = 968	Duration in seconds OR for each extra second taken to complete task	10.54 (4.57) *n* = 608	11.46 (6.63) *n* = 360	1.032	1.006	1.059	0.01
30 s Chair stand *n* = 1033	Number in 30 s OR for each additional chair stand	10.66 (3.25) *n* = 657	10.14 (3.37) *n* = 376	0.951	0.915	0.990	0.01
Quality of Life^b^ *n* = 999	Total score OR for each extra point	130.75 (13.23) *n* = 662	128.73 (13.30) *n* = 337	0.989	0.978	0.999	0.03
SF12_MCS^c^ *n* = 1050	Total score OR for each extra point	48.94 (5.85) *n* = 663	49.31 (6.31) *n* = 386	0.984	0.964	1.005	0.13
Functional reach *n* = 1017	Functional reach in cms OR for each additional cm reached	26.02 (7.97) *n* = 642	25.29 (8.02) *n* = 375	0.988	0.979	0.999	0.15
FICSIT^d^ *n* = 1057	Score 0–28 OR for each additional point scored	20.54 (6.84) *n* = 667	19.91 (7.33) *n* = 390	0.987	0.973	1.005	0.15
Dichotomised Variable	Category	Not sedentary *n* (%)	Sedentary *n* (%)	OR	Lower CI	Upper CI	*P*-value
Age *n* = 1052	65–74	428 (63.4)	247 (36.6)	1.024	0.788	1.329	0.86
	75 + years	237 (62.9)	140 (37.1)				
Gender *n* = 1053	Male	241 (61.5)	151 (38.5)	0.886	0.685	1.147	0.36
	Female	425 (64.3)	236 (35.7)				
BMI *n* = 1008	Normal	266 (72.9)	99 (27.1)	1.982	1.500	2.620	<0.001
	Abnormal	370 (57.5)	273 (42.5)				
Smoking *n* = 1053	Never	353 (67.5)	170 (32.5)	1.440	1.119	1.852	0.005
	Ever (ex/current)	313 (59.1)	217 (40.9)				
Living circumstances *n* = 1051	As a couple	405 (65.6)	212 (34.4)	1.291	1.002	1.664	0.05
	Not as a couple	259 (59.7)	175 (40.3)				
Informal home help *n* = 1047	Absence	625 (64.4)	346 (35.6)	2.007	1.256	3.208	0.003
	Presence	36 (47.4)	40 (52.6)				
Current activity level *n* = 1005	Some	392 (66.1)	201 (33.9)	1.356	1.046	1.759	0.02
	None	243 (59.0)	169 (41.0)				
Public transport use *n* = 1047	Yes (easy)	634 (64.0)	357 (36.0)	1.907	1.112	3.273	0.02
	No (not easy)	27 (48.2)	29 (51.8)				
Walking aid used? *n* = 1052	No	586 (65.0)	315 (35.0)	1.302	1.094	1.549	0.003
	Yes	79 (52.3)	72 (47.7)				
Employment status *n* = 1048	Employed	56 (62.9)	33 (37.1)	0.984	0.628	1.543	0.94
	Not employed	607 (63.3)	352 (36.7)				
Educational level *n* = 1038	FE & University	304 (64.3)	169 (35.7)	1.097	0.851	1.413	0.48
	School only	351 (62.1)	214 (37.9)				
Household income (categorical linear) *n* = 913	Up to £12,000	159 (56.8)	121 (43.2)	-	-	-	0.13
	£12,001–20,000	168 (64.1)	94 (35.9)	0.735	0.520	1.039	0.08
	£20,001–30,000	133 (65.5)	70 (34.5)	0.692	0.476	1.005	0.05
	£30,001–45,000	66 (69.5)	29 (30.5)	0.577	0.351	0.949	0.03
	>£45,001	44 (60.3)	29 (39.7)	0.866	0.512	1.464	0.59

Varying denominators reflect variations in data capture

^a^higher score = better physical function

^b^higher score = better self-rated quality of life

^c^higher score = better self-reported mental health

^d^higher score = better balance

In this study sample, sedentary behaviour was not significantly different between men and women, and was not more common amongst those aged 75 and over than those 65–74. SB was associated with having an abnormal BMI (<18.5 or >25) and 64 % of the sample had abnormal BMIs. Only 18 of the sample had BMI values below 18.5, but 44 % were sedentary; 42 % of the 625 participants with BMI >25 kg/m^2^ were sedentary.

Univariable analyses showed that those who were sedentary were more likely to: ever have smoked, have more comorbidities, take more medications, have difficulty using public transport, use a walking aid, not live in a couple, have informal home help, and describe themselves as inactive. The sedentary reported greater activity limitation, poorer quality of life and poorer physical health as well as performing less well on some functional tests; timed up and go & chair stand. There were no statistically significant associations between sedentary behaviour and educational attainment, household income, employment status, falls in the last year, falls risk (Falls Risk Assessment Tool, FRAT), functional reach, balance (FICSIT), self-reported mental health (SF12-mental component score (Mental Component Score, MCS) and social isolation (Lubben social network score). These variables were excluded from further analysis. Table 3 shows the associations between sedentariness and continuous variables, and dichotomised variables.

Logistic regression analyses showed that only 4 covariates remained independently significantly associated with sedentary behaviour:

1. Abnormal BMI (OR 1.740 CI 1.248–2.425, *p* = 0.001),
2. Ever smoked (OR 1.420, CI 1.043–1.934, *p* = 0.03),
3. Number of medications taken (OR 1.069, CI 1.016–1.124, *p* < 0.001),
4. Self-reported physical health (SF12-PCS) (OR 0.961, CI 0.933–0.990, *p* < 0.001).

For each additional medication the odds of being sedentary increased and for each additional point on the SF-12 PCS (indicating better self-rated health) the odds of being sedentary decreased.

## Discussion

### What this study shows

The offer of exercise promotion did not just attract an already healthy, active group of older people. Almost half (42 %) of older adults recruited to this intervention study were sedentary by our definition and could therefore benefit from increasing their activity levels. To our knowledge the characteristics of thisrgroup of sedentary older people who engaged with exercise promotion has not been examined previously on this scale in the UK.

Sedentary participants in our exercise programme were different to sedentary older people described by epidemiological studies. In our sample of older people joining an exercise trial sedentariness was not associated with age, education, income, gender or functional fitness; this is not surprising, given that trial participants tend to be healthier than the general population. In this sample four characteristics were associated with sedentary behaviour; abnormal BMI, smoking status, self-reported physical health and multiple medication use. Ever smoking, abnormal BMI, multiple medication usage and poor self-reported physical health are markers of poor health. Smoking predicts cardiovascular disease, whilst self-reported limitation is more predictive of future adverse outcomes including mortality than the objective number of comorbidities [40, 41]. Use of multiple medication use is not the same as having comorbidities, in this sample. Overall, the sedentary people in this trial were in poor health but nevertheless interested in increasing their physical activity.

### Strengths and limitations

Our study’s strengths include the large number of participants enrolled who are sedentary and our ability to characterise that cohort in detail. Additionally it was able to focus on sedentary behaviour even in those who were physically active.

We are limited by the secondary analysis of data from the pre-existing ProAct65+ trial. As such, our sedentary score is an estimate from the PASE questionnaire and not from a dedicated assessment tool for sedentariness, nor from an objective measurement. The trial excluded participants if they were frequent fallers or had poor mobility prior to the offer of participation in the exercise programme, making it likely that some of the most sedentary older adults were excluded from the trial. This self-selected population cannot be thought of as a typical of the wider population of sedentary older people, and we cannot estimate the number of sedentary and non-sedentary individuals among the 20,507 people initially invited to the trial. Any stepwise analysis could result in a combination of predictor variables that apply only to that dataset, and so such results need to be replicated in an independent dataset.

### Implications for practice and research

Our sedentary group had good grounds for wanting to increase their activity levels. They are already experiencing ill health as evidenced by their medication use, self-rated physical health, smoking status and abnormal BMI. This study suggests that some sedentary older people who would benefit from exercise promotion would join exercise promotion interventions organised through General Practice. Further investigation of the impact of exercise promotion on sedentariness is required as recent studies have had short follow ups [34–36]. Longer term studies examining the effect of exercise promotion on a group of sedentary older adults are required.

## Conclusion

Offering exercise opportunities to older people does attract some sedentary participants with a poor health profile (characterised above) who are likely to benefit from such intervention.

## Abbreviations

SB: Sedentary behaviour
PASE: Physical activity scale for the elderly
MVPA: Moderately-vigorous physical activity
FaME: Falls management exercise programme
OEP: Otago exercise programme
TAU: Treatment as usual
FICSIT: Frailty and Injuries: Cooperative Studies of Intervention Techniques
FRAT: Falls risk assessment tool
MCS: Mental component score
PCS: Physical component score
OPQOL: Older people’s quality of life questionnaire
TUG: Timed get-up and go test
FES-I: Falls efficacy scale-international
PHQ: Physical health questionnaire
BMI: Body mass index
